# Strategies to improve the therapeutic effects of mesenchymal stromal cells in respiratory diseases

**DOI:** 10.1186/s13287-018-0802-8

**Published:** 2018-02-26

**Authors:** Luisa H. A. Silva, Mariana A. Antunes, Claudia C. Dos Santos, Daniel J. Weiss, Fernanda F. Cruz, Patricia R. M. Rocco

**Affiliations:** 1 Laboratory of Pulmonary Investigation, Carlos Chagas Filho Institute of Biophysics, Federal University of Rio de Janeiro, Centro de Ciências da Saúde, Avenida Carlos Chagas Filho, s/n, Bloco G-014, Ilha do Fundão–, Rio de Janeiro, RJ 21941-902 Brazil; 2 National Institute of Science and Technology for Regenerative Medicine, Rio de Janeiro, Brazil; 3 The Keenan Research Centre for Biomedical Science of the Li Ka Shing Knowledge Institute of St. Michael’s Hospital, Toronto, ON Canada; 4 Department of Medicine, Vermont Lung Center, College of Medicine, University of Vermont, Burlington, USA

**Keywords:** Mesenchymal stromal cells, Hypoxia, Serum deprivation, Genetic manipulation

## Abstract

Due to their anti-inflammatory, antiapoptotic, antimicrobial, and antifibrotic properties, mesenchymal stromal cells (MSCs) have been considered a promising alternative for treatment of respiratory diseases. Nevertheless, even though MSC administration has been demonstrated to be safe in clinical trials, to date, few studies have shown evidence of MSC efficacy in respiratory diseases. The present review describes strategies to enhance the beneficial effects of MSCs, including preconditioning (under hypoxia, oxidative stress, heat shock, serum deprivation, and exposure to inflammatory biological samples) and genetic manipulation. These strategies can variably promote increases in MSC survival rates, by inducing expression of cytoprotective genes, as well as increase MSC potency by improving secretion of reparative factors. Furthermore, these strategies have been demonstrated to enhance the beneficial effects of MSCs in preclinical lung disease models. However, there is still a long way to go before such strategies can be translated from bench to bedside.

## Background

Mesenchymal stromal cells (MSCs) are at the forefront of the regenerative medicine field. By definition, human MSCs adhere to plastic when maintained in culture; express the CD105, CD90, and CD73 cell surface markers and lack CD45, CD34, and CD14; and differentiate *in vitro* into osteoblasts, adipocytes, and chondrocytes in the presence of inducers [1]. In the past decade, MSCs were also proven to have immunomodulatory properties [2]: they suppress proliferation, maturation, and differentiation of immune cells, such as macrophages, dendritic cells, and natural killer cells, as well as of B and T lymphocytes [2].

MSCs attenuate inflammation through different mechanisms, such as: 1) secretion of paracrine/endocrine mediators, including hormones, cytokines, growth factors, lipid mediators, mRNAs, and microRNAs (miRNAs), in extracellular vesicles or otherwise [2, 3]—these secreted factors can have a wide variety of anti-inflammatory, antiapoptotic, antimicrobial, and antifibrotic activities [2, 3]; 2) cell-to-cell contact, which exerts effects on immune cells through recognition of ligands to receptors [4]; and 3) transfer of organelles, such as mitochondria [4, 5].

**Fig. 1:**
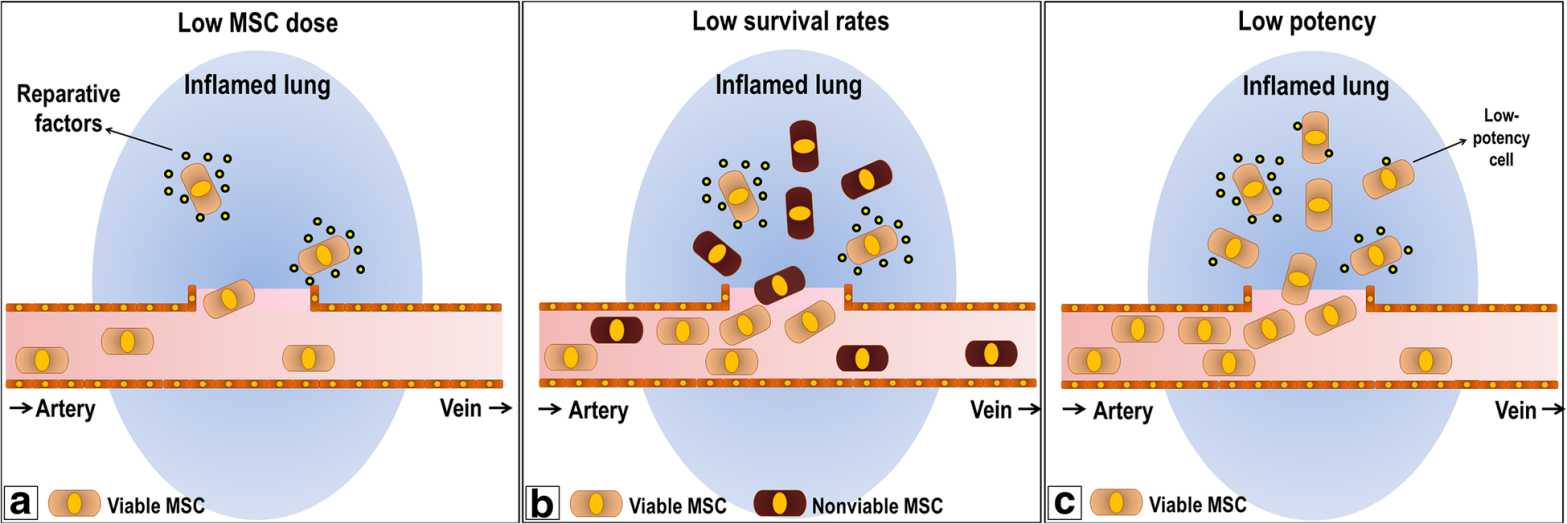
Factors that hinder MSC-based therapies. **a** The small amount of MSCs inoculated. Additionally, although MSCs are easily trapped in pulmonary capillaries after systemic administration, there is no long-term retention. Thus, the amount of restorative factors is progressively reduced. **b** MSCs are vulnerable to the toxicity of inflamed microenvironments, resulting in low survival rates *in vivo*. The few remaining viable MSCs might not be enough to exert adequate therapeutic effect. **c** Although viable and in adequate numbers, MSCs may still have low potency, i.e., lack activity or effectiveness to attenuate inflammation or repair injured tissue

Because of these mechanisms, MSCs have been assessed as potential therapies for lung diseases, such as acute respiratory distress syndrome (ARDS) [6], allergic asthma [7, 8], emphysema [9], and silicosis [10], and have been tested as such in experimental settings. The beneficial effects of MSCs in these preclinical studies have encouraged the initiation of clinical trials, which reported a good safety profile [11–13], even though the potential efficacy of MSC therapy was found to be limited [14, 15]. This limited efficacy may be due to several factors, including the small amount of MSCs inoculated (Fig. 1a) [15], MSC administration late in the course of lung disease [16, 17], low MSC survival rates *in vivo* (Fig. 1b) [18], and impaired MSC potency/biological activity (Fig. 1c) [2].

MSC engraftment in the lungs is another issue that hinders cell therapy. It is estimated that MSCs are cleared from lung tissue within 24 h [19]. Essentially, only two strategies have been tested to address this. In the first, overexpression of the surface receptor CXCR-4, which interacts with stromal cell-derived factor-1, supports MSC homing to injured sites [20]. Therefore, in acute lung injury models, more MSCs move into and settle in the lungs. In the second strategy, MSCs are recruited into the lung tissue by the magnetic targeting technique [21]. Although this technique improves cell retention after 48 h, its efficacy has yet to be evaluated.

Two strategies have been proposed to improve MSC survival or potency and thus enhance the beneficial effects of these cells. The first is preconditioning which is based on the biological concept of hormesis whereby brief exposure to low doses of an otherwise toxic or lethal agent leads to beneficial effects (stress tolerance growthor longevity) [22]. The other strategy is genetic manipulation. Genes involved in cell survival pathways and immunomodulation are modulated by plasmid transfection; by transduction with viral vectors; or by miRNA and small interfering RNA (siRNA).

The aim of the present review is to describe and discuss the strategies above and how they have contributed to advancing the treatment of pulmonary diseases in the experimental setting.

## Strategies to improve MSC survival rate

The success of MSC therapy requires an appropriate number of cells. For this purpose, MSCs are expanded *ex vivo*, in culture medium containing animal sera rich in growth factors. Nevertheless, successive replications, culture conditions, and freezing/thawing may be deleterious to the cells, rendering them more susceptible to the hostile environment of injured tissue [23, 24].

Recent data suggest that freshly thawed MSCs may not have the same effectiveness or breadth of anti-inflammatory activities as do freshly cultured MSCs [24]. Conversely, our group recently reported that thawed MSCs are as effective as freshly cultured MSCs in experimental allergic asthma [25]. Thus, further  studies are required to evaluate fresh *versus* thawed MSC effectiveness in different *in vivo*, in disease-specific models.

**Fig. 2:**
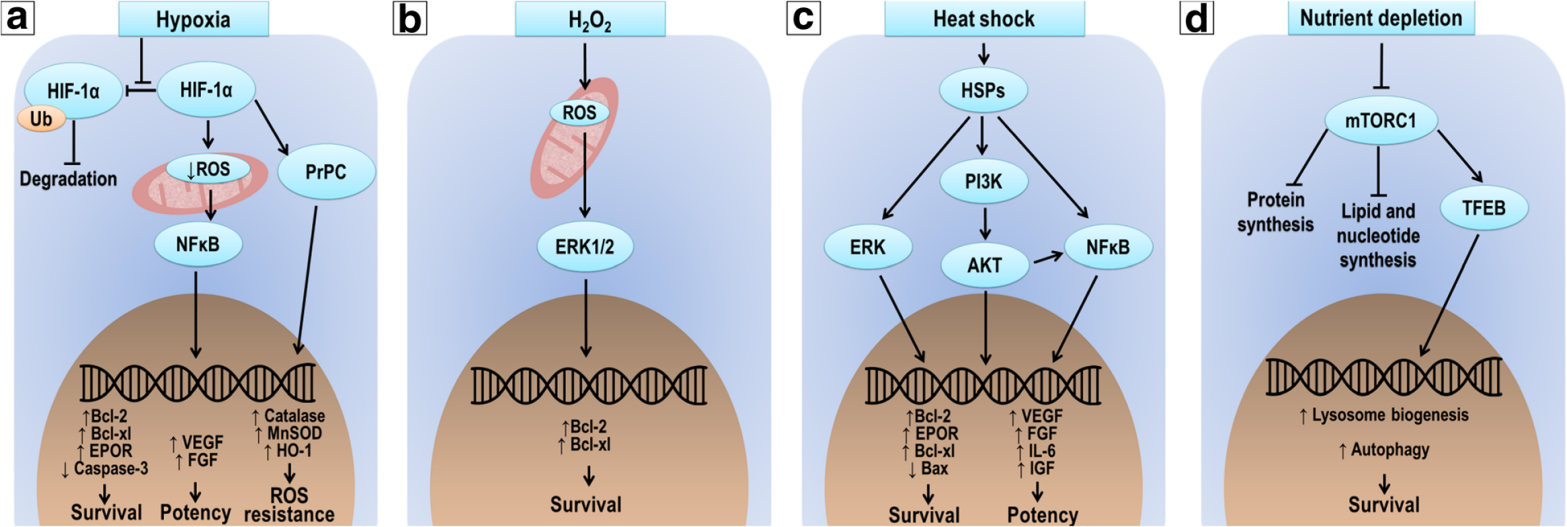
Preconditioning strategies to improve MSC survival. **a** Hypoxic preconditioning promotes stabilization of hypoxia-inducible factor 1-α (*HIF-1α*), which decreases reactive oxygen species (*ROS*) levels in MSC mitochondria, activating nuclear factor kappa B (*NFκB*). HIF-1α also stimulates synthesis of normal cellular prion protein (*PrPC*). NFκB and PrPC promote expression of anti-apoptotic proteins, repairing growth factors, and antioxidant enzymes. **b** Oxidative preconditioning raises ROS levels in MSC mitochondria, activating extracellular signal-regulated kinases (*ERK*), leading to expression of genes involved with survival. **c** Heat shock preconditioning leads MSCs to produce heat shock proteins (*HSPs*), which promote survival and potentiation through three different signaling pathways: ERK, PI3K/AKT, and NFκB. **d** Nutrient-depletion preconditioning inhibits mammalian target of rapamycin complex-1 (*mTORC1*), interrupting protein, lipid, and nucleotide synthesis. On the other hand, inhibition of mTORC1 favors the activity of proteins such as transcription factor EB (*TFEB*), which promotes expression of genes related to lysosomal biogenesis and leads to protective autophagic processes

Preconditioning strategies have been tested to protect MSCs from injured environments, thus increasing their survival. Some research has focused on the response of MSCs to sublethal exposure to cellular stressors, such as hypoxia, heat, and nutrient depletion (Fig. 2), which resemble either ischemic or inflammatory microenvironments and are considered the major challenges to cell survival *in vivo* [22, 26].

Besides preconditioning, manipulation of genes involved in vital cell cycles, apoptosis, and cell survival pathways has also been tested in an attempt to increase MSC survival rates [26]. For example, MSCs overexpressing hepatocyte growth factor (HGF) exhibit improved survival *in vivo*, and their effects have already been tested in lung injury models [27, 28].

### Preconditioning strategies to improve MSC survival

#### Hypoxic preconditioning

Oxygen concentration in MSC niches is 10–15% in adipose tissue, 1–7% in bone marrow, and 1.5–5% in female reproductive tract and birth-associated tissues [29]. Because of the greater oxygen supply during *ex vivo* culture (usually 20% O_2_), MSCs are susceptible to oxidative stress, affecting their viability [29, 30]. However, when cultured under hypoxia, compared to normoxia, fewer MSCs express senescence-associated β-galactosidase (≅10% at hypoxia *vs* ≅45% at normoxia) [31] and caspase-3 (25.6 ± 5.4% *vs* 36.6 ± 6.6%) [32]. Additionally, MSCs under hypoxia exhibit more population doubling (37.5 ± 3.4 *vs* 28.5 ± 3.8) [31].

Stabilization of hypoxia-inducible factor (HIF)-1α can explain these findings (Fig. 2a). HIF-1α levels in MSCs are increased 3.4-fold after 24 h at 0.5% O_2,_ indicating that it is normally degraded under normoxic conditions [22, 32]. In MSCs, HIF-1α alters energy metabolism, blocks oxidative phosphorylation, and promotes glycolysis, thus reducing reactive oxygen species (ROS) production [22]. This activates nuclear factor kappa B (NF-κB), which upregulates antioxidant and antiapoptotic proteins [22, 32]. Exposure to 0.5% O_2_ for 24 h led to increases in Bcl (B-cell lymphoma)-X_L_ (≅1.6-fold) and Bcl-2 (≅1.25-fold) levels, as well as to a decrease in caspase-3 (≅0.7-fold) levels [22]. Recently, HIF-1α was also found to activate normal cellular prion protein (PrPC), which increases activity of superoxide dismutase (SOD) and catalase, protecting MSCs against oxidative stress [33].

Therefore, hypoxic preconditioning prepares MSCs for survival in ischemic microenvironments, with promising results for treatment of lung diseases associated with gas-exchange impairment (ARDS, emphysema, asthma, and pulmonary fibrosis). In a model of pulmonary fibrosis, compared to MSCs cultured under normoxia (NP-MSCs), hypoxia-preconditioned MSCs (HP-MSCs) attenuated bleomycin-induced airway constriction (Penh values 0.7 ± 0.07 (HP-MSCs) *vs* 1.19 ± 0.34 (NP-MSC)) and lung edema (wet-to-dry ratio 0.32 ± 0.05 *vs* 0.43 ± 0.05) to a greater extent [34]. HP-MSCs were also more effective at attenuating fibrotic changes compared to NP-MSCs (Ashcroft scores 2.13 ± 0.81 vs 3.9 ± 0.57) [34]. In this report, cell survival was evaluated using MSC transduction with the lacZ reporter gene. The HP-MSC treatment group exhibited three times more lacZ mRNA in lung tissue after 18 days compared to the NP-MSC group. The authors suggest that improvement in lung function and histology may be associated with the use of hypoxic preconditioning, which increases MSC survival.

#### Preconditioning through oxidative stress exposure, heat shock protein, and serum deprivation

MSCs preconditioned by exposure to oxidative stress, heat shock protein, and serum deprivation have not yet been tested in lung disease models. However, results of *in vitro* and *in vivo* studies support the use of these strategies.

For example, oxidative preconditioning promotes not only a cytoprotective effect, but also an increase in MSC potency. MSCs previously exposed to non-lethal H_2_O_2_ concentrations (20 and 50 μM) were more resistant against a lethal dose of this compound [35]. In the control group (0 μM), ≅60% of MSCs were found to be in apoptosis, *versus* ≅30% and ≅40% in the 20 and 50 μM groups, respectively [35]. The molecular mechanism involved in cytoprotection is a transient release of ROS by MSC mitochondria, which activates extracellular signal-regulated kinases (ERKs) [22, 35]. The ERK pathway promotes the expression of antiapoptotic proteins, such as Bcl-2 and Bcl-X_L_ (Fig. 2b) [22, 35]. This strategy may be interesting for treatment of inflammatory diseases that feature ROS release in pulmonary microenvironments, such as ARDS and silicosis.

Heat-shock preconditioning has also emerged as an interesting approach to increase MSC survival rates. Exposure of MSCs to 42 °C for 60 min led to increased expression of HSP-27 and HSP-90 (4.8-fold and 17.4-fold, respectively) [36]. These HSPs contribute to MSC viability by activating the phosphoinositide 3-kinase (PI3K/AKT), ERK, and NF-κB signaling pathways (Fig. 2c) [22]. Indeed, MSCs cultured with exogenous HSP90α exhibited elevated expression of Bcl-2 and Bcl-X_L_ proteins, blunted expression of Bax (a member of the Bcl-2 gene family), and cleaved caspase-3 proteins [37]. Despite the potential applications, heat shock preconditioned-MSCs have never been tested *in vivo*.

Lastly, serum depletion (SD) preconditioning aims to reduce MSC energy demand by keeping them in a quiescent state, which would facilitate their survival in ischemic environments [38]. In a recent report, MSCs were cultured without fetal bovine serum for 48 h. Constructs containing these MSCs were then implanted in animals and maintained in ischemic conditions for 3 and 7 days. Constructs were then explanted, MSCs were isolated from the scaffold, and flow cytometry (propidium iodide staining) was performed for viability measurement. The SD-preconditioned group exhibited four times and three times more viable MSCs, on days 3 and 7, respectively, compared with the control group [38].

During SD preconditioning, the absence of growth factors inhibits the mammalian target of rapamycin (mTOR) signaling pathway, more specifically mTOR complex-1 (mTORC1) [38]. Among other functions, mTORC1 suppresses catabolic processes such as autophagy [38, 39]. Indeed, after 48 h of SD preconditioning, MSCs exhibited higher levels of endogenous LC3B-II protein (an autophagosome marker) compared to control cells (68 *vs* 38% of LC3-positive MSCs) [38]. Importantly, when autophagy was inhibited, half of MSCs died after 7 days of culture under ischemia. Therefore, the enhanced survival of MSCs thus preconditioned may be due to a protective autophagy process [38].

#### Genetic manipulation to improve MSC survival

Several genetic approaches have been used to improve MSC survival [26]. Although most of these approaches have aimed to improve treatment of myocardial infarction—by making MSCs more resistant to ischemic environments—HGF-overexpressing MSCs (HGF-MSCs) have also been tested in acute lung injury models. HGF is a growth factor with anti-inflammatory, antiapoptotic, and reparative properties [28].

After *in vitro* exposure to H_2_O_2_ (120 μM) for 4 h, HGF-MSCs presented a lower apoptosis rate compared to unmodified cells (25.3 vs 64.6% annexin-V positive cells). The number of apoptotic cells *in vivo* fell by almost half after HGF overexpression [28]. Nevertheless, the mechanisms by which HGF increases MSC survival require elucidation.

HGF upregulation also increased MSC potency. HGF-MSCs increased HGF levels in lung tissue, and, compared to wild-type MSCs, they improved oxygenation (PaO_2_ levels 104.62 ± 10.5 *vs* 90.30 ± 8.8 mmHg), decreased lung injury scores (0.3 *vs* 0.55) and neutrophil infiltration (MPO activity ≅600 *vs* ≅700 mU/g), increased SOD levels, and upregulated IL-10 (≅4-fold *vs* 2-fold increase) [28].

Therefore, several strategies have been tested to improve MSC survival *in vivo*. Nevertheless, it bears stressing that some reports suggest that even MSCs undergoing apoptosis have immunomodulatory activity. In asthma models, administration of 10^6^ apoptotic MSCs exerted an immunosuppressive effect, significantly reducing eosinophil infiltration in BAL by half, compared to viable MSCs and no treatment [40]. In a model of sepsis, therapy with apoptotic MSCs improved arterial oxygen saturation and reduced lung damage (lung weight to body weight ratio ≅0.45 × 10^−2^ vs ≅ 0.4 × 10^−2^) [41]. However, the mechanism of action by which apoptotic MSCs improve therapy outcomes remains unclear.

## Strategies to improve MSC potency

MSC “potency” is defined as a measure of its biological activity; it is also a relationship between therapeutic effects and the MSC dose required to achieve them [2]. Low MSC potency may be a result of MSC phenotype changes during *in vitro* expansion [23]. In addition, MSCs are not spontaneously immunosuppressive; prior activation is required to increase their potency [2]. Therefore, it is important to understand the mechanisms that lead to MSC activation.

### Preconditioning strategies to improve MSC potency

#### Preconditioning in an inflammatory milieu

In a microenvironment undergoing inflammation, macrophages and neutrophils release pro-inflammatory mediators (interferon (IFN)-γ, TNF-α, IL-1, chemokines, leukotrienes, and free radicals). MSCs “sense” these mediators and activate NF-κB, which promotes increased expression of immunomodulatory and repair factors [2, 42].

Other factors also activate MSCs. Microbe-associated molecular patterns, for instance, do so through Toll-like receptors (TLRs), such as TLR3 and TLR4. Activation through TLR4 with lipopolysaccharide (LPS) induces a pro-inflammatory MSC phenotype, with secretion of IL-6, IL-8, and transforming growth factor (TGF)-β. Otherwise, activation through TLR3 with polyinosinic:polycytidylic acid (poly I:C) induces an anti-inflammatory MSC phenotype, with expression of indoleamine 2,3-dioxygenase, prostaglandin (PG)E2, IL-4, and IL-1RA [2].

Based on the foregoing, the first strategy to improve MSC potency is preconditioning in an inflammatory milieu. In the context of lung diseases, pooled serum from patients with moderate to severe ARDS has been used as an inflammatory background to activate MSCs [42]. This serum contains high levels of IL-10, IL-8, and IL-6, as well as low levels of IL-1β, TNF-α, and IFN-γ. When preconditioned with 0.5% ARDS serum for 16 h, MSCs exhibited significantly increased expression of IL-10 (≅5-fold) and interleukin-1 receptor antagonist (IL-1RN; ≅2.5-fold) compared to control MSCs. ARDS serum also reduced expression of pro-inflammatory mediators (Table 1) [42].

**Table 1:** Preconditioning strategies to improve MSC potency in lung diseases

Preconditioning strategy	Human MSC source	*In vitro* effects (compared to naïve MSCs)	In vivo effects (compared to naïve MSCs)	Lung disease model	Reference
ARDS serum(0.5%; 16 h)	Bone marrow	↑ IL-10 and IL-1RN mRNA expression/protein levels↓ IL-6, IL-8, IL-1α, IL-1β, IFN-γ, TGF-β2, and β3 expression/levels	↓ Inflammatory cells in BALF; histological lung scores; lung vascular permeability ↑ IL-10 levels in plasma and BALF ↓ IL-6 and IL-8 levels in plasma ↓ IL-1β and TNF-α levels in BALF	LPS-induced ARDS	[42]
Pioglitazone (3 μmol/L; 1 week)	Adipose tissue	↑ VEGF protein levels ↑ Stimulation of murine lung epithelial cell proliferation	↑ FGF-2, VEGF, and HGF protein levels in lung homogenate ↓ Morphometric changes	Smoke-induced emphysema	[43]
N-acetylcysteine (2 mM; 24 h)	Embryonic tissues	↑ Intracellular glutathione content ↓ ROS levels	↓ Lung injury score; collagen deposition; inflammatory cells in BALF; and apoptotic lung cells ↓ IL-6, TNF-α, and IL-1β protein levels in BALF ↑ Survival rates	Bleomycin-induced lung injury	[44]
Tetrandrine (5 and 10 μM; 24 h)	Bone marrow	PGE-2 activation ↓ TNF-α secretion by LPS-activated macrophages	–	–	[45]

*BALF* bronchoalveolar lavage fluid, *FGF* fibroblast growth factor, *VEGF* vascular endothelial growth factor

In a model of *Escherichia coli* LPS-induced lung injury, MSCs activated with ARDS serum were more effective than naïve cells at increasing IL-10 levels (100-fold in plasma and twofold in bronchoalveolar lavage fluid (BALF)) and reducing inflammatory cell counts (≅15 × 10^4^ *vs* ≅60 × 10^4^) and inflammatory cytokines in BALF, as well as lung inflammation score and vascular permeability (Table 1) [42].

#### Preconditioning with other substances

There is interest in exploring alternative methods to increase MSC potency, and recent studies have addressed the combination of MSCs and other substances . These have included pioglitazone [43], *N*-acetylcysteine [44], and tetrandrine [45] (Table 1).

Pioglitazone is an antidiabetic drug that binds to peroxisome proliferator-activated receptor (PPAR)-γ, modulating transcription of genes involved in glucose and lipid metabolism [43]. MSC treatment with pioglitazone (3 μmol/L for 1 week) led to increased vascular endothelial growth factor (VEGF) expression, thus improving murine lung epithelial cell proliferation *in vitro* compared to control MSCs. Administration of these pioglitazone-preconditioned MSCs in a cigarette smoke-induced emphysema model enhanced levels of reparative factors in lung tissue (Table 1) and attenuated lung morphometric changes (mean linear intercepts 75.6 ± 1.4 with preconditioned *vs* 80.5 ± 3.2 μm with control MSCs). It is worth noting that the precise mechanisms of action of pioglitazone on MSCs have yet to be elucidated [43].

The mucolytic agent *N*-acetylcysteine (NAC) has been tested for lung injury treatment because of its antioxidant effect [44]. Pretreatment with NAC (2 mM for 24 h) improved MSC antioxidant capacity *in vitro* by restoring glutathione levels (≅100% increase compared to non-activated MSCs). In a bleomycin-induced lung injury model, compared to non-activated cells, NAC-preconditioned MSCs reduced lung inflammation and collagen content in pulmonary tissue (Table 1) [44]. As a result, treatment with primed MSCs significantly reduced mortality 28 days after bleomycin administration compared to treatment with naïve MSCs or no treatment (83.3 *vs* 60 and 40%, respectively) [44].

MSCs preconditioned with tetrandrine—an alkaloid originally isolated from the Chinese medicinal herb *Stephania tetrandra*—have not yet been tested for treatment of lung diseases. However, an *in vitro* study reported that exposure to tetrandrine (5 and 10 μM for 24 h) increased PGE2 expression in MSCs. *In vitro,* activated MSCs attenuated TNF-α secretion by LPS-stimulated RAW264.7 macrophages by 25% compared to naïve cells [45] (Table 1). These results demonstrate the potential of tetrandrine-primed MSCs as therapeutic agents for lung diseases.

#### MSC potentiation by genetic manipulation

A variety of mitogenic antiapoptotic and anti-inflammatory factors have been quite efficiently transduced into MSCs. These manipulated cells have been tested in experimental models of ARDS pulmonary hypertension (PAH) and chronic obstructive pulmonary disease.

ARDS has been a frequent target of this technique. In an attempt to ensure effective reversal of the inflammatory process, MSCs have been transduced with the following genes: developmental endothelial locus-1 (Del-1), a glycoprotein secreted by endothelial cells that plays critical roles in cell migration and infiltration [46]; ST2 receptor gene (sST2), a catch receptor for IL-33—the IL-33–ST2 axis bridges innate and adaptive immune responses during lung inflammation [47]; angiotensin-converting enzyme-2 (ACE-2) [48], an enzyme that reduces levels of Ang-2, an essential mediator of ARDS pathogenesis; and manganese superoxide dismutase (MnSOD) [49], an enzyme that protects mitochondria against ROS.

When tested in murine models of ARDS, MSCs transduced with these genes significantly reduced lung injury index [46, 48, 49], neutrophil count [46–49], levels of pro-inflammatory cytokines (TNF-α, IL-β, and/or IL-6) [46–49], and protein content [46, 47] in bronchoalveolar lavage fluid (BALF). In addition, some of these MSCs increased levels of the anti-inflammatory cytokine IL-10 [47–49] and reduced pulmonary edema [46, 49] and the apoptosis rate [49] in pulmonary tissue, improving survival in mice [49] (summarized in Table 2).

**Table 2:** Gene therapy approaches to improve MSC potency in lung diseases

Lung disease model	Upregulated gene	MSC source	MSC dose	Time of MSC administration	In vivo effects (compared to wild-type MSC)	Reference
LPS-induced ARDS	Developmental endothelial locus-1	Murine bone marrow	5 × 10^6^	1 h after LPS injection	**↓** Lung injury histopathological index **↓** Pulmonary edema **↓** Neutrophil counts, TNF-α levels, and protein concentration in BALF **↓** Myeloperoxidase activity in lung homogenates	[46]
	ST2 receptor gene	Human adipose tissue	10^6^	6 h after LPS injection	**↑** IL-10 mRNA levels in lung homogenate **↓** IL-1β and IFN-γ mRNA levels in lung homogenate **↓** LPS-mediated production of circulating IL-33 **↓** TNF-α and IL-6 levels and protein concentration in BALF **↓** Polymorphonuclear cells in interstitial space	[47]
	Angiotensin-converting enzyme-2	Murine bone marrow	5 × 10^5^	4 h after LPS injection	**↓** Lung injury histopathological index **↓** Total cell counts in BALF **↓** Neutrophil counts in BALF **↓** Ang-2, IL-1β and IL-6 protein levels in lung homogenates **↑** IL-10 protein levels in lung homogenates **↓** IL-1β serum levels **↓** Vascular permeability	[48]
Radiation-induced ARDS	Manganese superoxide dismutase	Human bone marrow	10^6^	4 h after exposure to radiation	**↓** Lung injury histopathological index **↓** Pulmonary edema **↓** TNF-α and IL-6 serum levels **↑** IL-10 serum levels **↓** Hydroxyproline in lung homogenates **↓** Neutrophil counts in BALF **↓** Lipid peroxidation **↓** Cell apoptosis in lung tissue **↑** Survival rates	[49]
Hypoxia-induced pulmonary hypertension	Heme oxygenase-1 isoform	Murine bone marrow	10^6^	2 weeks after exposure to hypoxia	**↓** Right ventricle systolic pressure **↓** Right ventricle hypertrophy	[50]

For the treatment of PAH, MSCs overexpressing the heme oxygenase isoform 1 (HO-1) gene have been tested. HO-1 protects cells against oxidative injury and contributes to regulation of vascular tone and smooth muscle proliferation [50]. MSCs isolated from transgenic mice harboring a human HO-1 transgene under the control of surfactant protein C promoter (HO-MSCs) were more effective in reducing RV systolic pressure (≅25 *vs* ≅32 mmHg) and RV hypertrophy (≅0.25 *vs* ≅0.28 RV/LV + S weight ratio) in wild-type recipients [50]. These results were not observed in HO-1 knockout mice, highlighting the role of endogenous HO-1 activity in protecting the lungs.

Preclinical studies revealed that MSCs have limited benefit in pulmonary emphysema. Genetically modified MSCs (HSP-VEGFA-MSC) with *cis*-resveratrol (c-RSV)-induced HSP70 promoter-regulated VEGFA expression have been evaluated in elastase-induced pulmonary emphysema in mice [51]. Intravenous administration of these HSP-VEGFA-MSCs led to significant improvement in respiratory function and lung histology in this emphysema model [51].

Each of these factors (Del-1, sST2, ACE-2, MnSOD, HO-1, HSP-VEGFA) has been found to aid lung tissue repair when administered in experimental models. Therefore, engineered MSCs that overexpress these factors present a synergistic mechanism of action. In other words, these cells simultaneously secrete paracrine immunomodulatory factors and promote a transient increase in lung levels of the cited proteins, thus potentiating the effects of cell therapy.

Importantly, the application of genetically modified stromal cells in the clinical setting is an imminent reality. Genetically modified cells have been approved and are now being used in early-phase clinical studies for patients with pulmonary hypertension, such as the Pulmonary Hypertension and Angiogenic Cell Therapy (PHACeT) trial [52].

## Conclusions

MSCs have potential in the regenerative medicine field. Nevertheless, the major outcomes of clinical trials of MSCs in respiratory disorders have fallen far short of the theoretical potential of these cells in preclinical studies. Transforming MSC transplantation into an efficient procedure is a huge challenge. Researchers have sought alternative and efficient strategies to improve the survival and immunomodulatory capacity of implanted MSCs and thus enhance tissue repair. However, despite a large body of experimental evidence for an arsenal of strategies to improve MSC function, as presented in this review, there is still a long way to go before such techniques can translate from bench to bedside.

## Abbreviations

AKT: Protein kinase B
ARDS: Acute respiratory distress syndrome
BALF: Bronchoalveolar lavage fluid
Bcl-2: B-cell lymphoma-2
ERK: Extracellular signal-regulated kinase
FGF: Fibroblast growth factor
HGF: Hepatocyte growth factor
HIF: Hypoxia-inducible factor
HO-1: Inducible heme oxygenase isoform 1
HSP: Heat shock protein
IFN: Interferon
IGF: Insulin-like growth factor
IL: Interleukin
LPS: Lipopolysaccharide
miRNA: MicroRNA
MnSOD: Manganese superoxide dismutase
MSC: Mesenchymal stromal cell
mTOR: Mammalian target of rapamycin
mTORC1: Mammalian target of rapamycin complex-1
NAC: N-acetylcysteine
NF-κB: Nuclear factor kappa B
PAH: Pulmonary hypertension
PG: Prostaglandin
PI3K: Phosphoinositide 3-kinase
PrPC: Normal cellular prion protein
ROS: Reactive oxygen species
SD: Serum depletion
SOD: Superoxide dismutase
TLR: Toll-like receptor
TGF: Transforming growth factor
TNF: Tumor necrosis factor
VEGF: Vascular endothelial growth factor

## References

[1] Dominici M, Le Blanc K, Mueller I, Slaper-Cortenbach I, Marini F, Krause D, Deans R, Keating A, Prockop D, Horwitz E (2006). Minimal criteria for defining multipotent mesenchymal stromal cells. The International Society for Cellular Therapy position statement. Cytotherapy..

[2] Ryan A, Murphy M, Barry F. Mesenchymal stem/stromal cell therapy. In: Atkinson K, editor. The Biology and therapeutic application of mesenchymal cells. Hoboken: Wiley; 2017. p. 426–40.

[3] Abreu SC, Weiss DJ, Rocco PRM (2016). Extracellular vesicles derived from mesenchymal stromal cells: a therapeutic option in respiratory diseases?. Stem Cell Res Ther..

[4] Spees JL, Lee RH, Gregory CA (2016). Mechanisms of mesenchymal stem/stromal cell function. Stem Cell Res Ther..

[5] Sinclair KA, Yerkovich ST, Hopkins PM-A, Chambers DC (2016). Characterization of intercellular communication and mitochondrial donation by mesenchymal stromal cells derived from the human lung. Stem Cell Res Ther..

[6] Maron-Gutierrez T, Silva JD, Asensi KD, Bakker-Abreu I, Shan Y, Diaz BL, Goldenberg RCS, Mei SHJ, Stewart DJ, Morales MM, Rocco PRM, Dos Santos CC (2013). Effects of mesenchymal stem cell therapy on the time course of pulmonary remodeling depend on the etiology of lung injury in mice. Crit Care Med..

[7] Cruz FF, Borg ZD, Goodwin M, Sokocevic D, Wagner DE, Coffey A, Antunes M, Robinson KL, Mitsialis SA, Kourembanas S, Thane K, Hoffman AM, McKenna DH, Rocco PRM, Weiss DJ (2015). Systemic administration of human bone marrow-derived mesenchymal stromal cell extracellular vesicles ameliorates Aspergillus hyphal extract-induced allergic airway inflammation in immunocompetent mice. Stem Cells Transl Med..

[8] Abreu SC, Antunes MA, Xisto DG, Cruz FF, Branco VC, Bandeira E, Kitoko J, de Araújo AF, Dellatorre-Texeira L, Olsen PC, Weiss DJ, Diaz BL, Morales MM, Rocco PR (2017). Bone marrow, adipose, and lung tissue-derived murine mesenchymal stromal cells release different mediators and differentially affect airway and lung parenchyma in experimental asthma. Stem Cells Transl Med.

[9] Antunes MA, Abreu SC, Cruz FF, Teixeira AC, Lopes-Pacheco M, Bandeira E, Olsen PC, Diaz BL, Takyia CM, Freitas IP, Rocha NN, Capelozzi VL, Xisto DG, Weiss DJ, Morales MM, Rocco PR (2014). Effects of different mesenchymal stromal cell sources and delivery routes in experimental emphysema. Respir Res..

[10] Lassance RM, Prota LFM, Maron-Gutierrez T, Garcia CSNB, Abreu SC, Pássaro CP, Xisto DG, Castiglione RC, Carreira H, Ornellas DS, Santana MCE, Souza SAL, Gutfilen B, Fonseca LMB, Rocco PRM, Morales MM (2009). Intratracheal instillation of bone marrow-derived cell in an experimental model of silicosis. Respir Physiol Neurobiol..

[11] Weiss DJ, Casaburi R, Flannery R, LeRoux-Williams M, Tashkin DP (2013). A placebo-controlled, randomized trial of mesenchymal stem cells in COPD. Chest..

[12] Morales MM, Souza SAL, Loivos LP, Lima MA, Szklo A, Vairo L, Brunswick THK, Gutfilen B, Lopes-Pacheco M, Araújo AJ, Cardoso AP, Goldenberg RC, Rocco PRM, Fonseca LMB (2015). Lapa e Silva JR. Pilot safety study of intrabronchial instillation of bone marrow-derived mononuclear cells in patients with silicosis. BMC Pulm Med..

[13] Stolk J, Broekman W, Mauad T, Zwaginga JJ, Roelofs H, Fibbe WE, Oostendorp J, Bajema I, Versteegh MIM, Taube C, Hiemstra PS (2016). A phase I study for intravenous autologous mesenchymal stromal cell administration to patients with severe emphysema. QJM..

[14] Zheng G, Huang L, Tong H, Shu Q, Hu Y, Ge M, Deng K, Zhang L, Zou B, Cheng B, Xu J (2014). Treatment of acute respiratory distress syndrome with allogeneic adipose-derived mesenchymal stem cells: a randomized, placebo-controlled pilot study. Respir Res..

[15] Wilson JG, Liu KD, Zhuo H, Caballero L, McMillan M, Fang X, Cosgrove K, Vojnik R, Calfee CS, Lee J-W, Rogers AJ, Levitt J, Wiener-Kronish J, Bajwa EK, Leavitt A, McKenna D, Thompson BT, Matthay MA (2015). Mesenchymal stem (stromal) cells for treatment of ARDS: a phase 1 clinical trial. Lancet Respir Med..

[16] McIntyre LA, Moher D, Fergusson DA, Sullivan KJ, Mei SHJ, Lalu M, Marshall J, Mcleod M, Griffin G, Grimshaw J, Turgeon A, Avey MT, Rudnicki MA, Jazi M, Fishman J, Stewart DJ (2016). Canadian Critical Care Translational Biology Group. Efficacy of mesenchymal stromal cell therapy for acute lung injury in preclinical animal models: a systematic review. PLoS One..

[17] Mei SHJ, Dos Santos CC, Stewart DJ (2016). Advances in stem cell and cell-based gene therapy approaches for experimental acute lung injury: a review of preclinical studies. Hum Gene Ther..

[18] Eggenhofer E, Benseler V, Kroemer A, Popp FC, Geissler EK, Schlitt HJ, Baan CC, Dahlke MH, Hoogduijn MJ (2012). Mesenchymal stem cells are short-lived and do not migrate beyond the lungs after intravenous infusion. Front Immunol..

[19] Wang H, Cao F, De A, Cao Y, Contag C, Gambhir SS, Wu JC, Chen X (2009). Trafficking mesenchymal stem cell engraftment and differentiation in tumor-bearing mice by bioluminescence imaging. Stem Cells..

[20] Yang J-X, Zhang N, Wang H-W, Gao P, Yang Q-P, Wen Q-P (2015). CXCR4 Receptor overexpression in mesenchymal stem cells facilitates treatment of acute lung injury in rats. J Biol Chem..

[21] Silva LHA, da Silva JR, Ferreira GA, Silva RC, Lima ECD, Azevedo RB, Oliveira DM (2016). Labeling mesenchymal cells with DMSA-coated gold and iron oxide nanoparticles: assessment of biocompatibility and potential applications. J Nanobiotechnology..

[22] Sart S, Ma T, Li Y (2014). Preconditioning stem cells for in vivo delivery. Biores Open Access..

[23] Lee KA, Shim W, Paik MJ, Lee SC, Shin JY, Ahn YH, Park K, Kim JH, Choi S, Lee G (2009). Analysis of changes in the viability and gene expression profiles of human mesenchymal stromal cells over time. Cytotherapy..

[24] François M, Copland IB, Yuan S, Romieu-Mourez R, Waller EK, Galipeau J (2012). Cryopreserved mesenchymal stromal cells display impaired immunosuppressive properties as a result of heat-shock response and impaired interferon-γ licensing. Cytotherapy..

[25] Cruz FF, Borg ZD, Goodwin M, Sokocevic D, Wagner D, McKenna DH, Rocco PRM, Weiss DJ (2015). Freshly thawed and continuously cultured human bone marrow-derived mesenchymal stromal cells comparably ameliorate allergic airways inflammation in immunocompetent mice. Stem Cells Transl Med..

[26] Pei M (2017). Environmental preconditioning rejuvenates adult stem cells’ proliferation and chondrogenic potential. Biomaterials..

[27] Wang H, Yang Y-F, Zhao L, Xiao F-J, Zhang Q-W, Wen M-L, Wu C-T, Peng R-Y, Wang L-S (2013). Hepatocyte growth factor gene-modified mesenchymal stem cells reduce radiation-induced lung injury. Hum Gene Ther..

[28] Chen S, Chen X, Wu X, Wei S, Han W, Lin J, Kang M, Chen L (2016). Hepatocyte growth factor-modified mesenchymal stem cells improve ischemia/reperfusion-induced acute lung injury in rats. Gene Ther.

[29] Amiri F, Jahanian-Najafabadi A, Roudkenar MH (2015). In vitro augmentation of mesenchymal stem cells viability in stressful microenvironments. Cell Stress Chaperones..

[30] Boregowda SV, Krishnappa V, Chambers JW, Lograsso PV, Lai W-T, Ortiz LA, Phinney DG (2012). Atmospheric oxygen inhibits growth and differentiation of marrow-derived mouse mesenchymal stem cells via a p53-dependent mechanism: implications for long-term culture expansion. Stem Cells..

[31] Fehrer C, Brunauer R, Laschober G, Unterluggauer H, Reitinger S, Kloss F, Gülly C, Gaßner R, Lepperdinger G (2007). Reduced oxygen tension attenuates differentiation capacity of human mesenchymal stem cells and prolongs their lifespan. Aging Cell..

[32] Hu X, Yu SP, Fraser JL, Lu Z, Ogle ME, Wang J-A, Wei L (2008). Transplantation of hypoxia-preconditioned mesenchymal stem cells improves infarcted heart function via enhanced survival of implanted cells and angiogenesis. J Thorac Cardiovasc Surg..

[33] Han Y-S, Lee JH, Yoon YM, Yun CW, Noh H, Lee SH (2016). Hypoxia-induced expression of cellular prion protein improves the therapeutic potential of mesenchymal stem cells. Cell Death Dis..

[34] Lan Y-W, Choo K-B, Chen C-M, Hung T-H, Chen Y-B, Hsieh C-H, Kuo H-P, Chong K-Y (2015). Hypoxia-preconditioned mesenchymal stem cells attenuate bleomycin-induced pulmonary fibrosis. Stem Cell Res Ther..

[35] Li S, Deng Y, Feng J, Ye W (2009). Oxidative preconditioning promotes bone marrow mesenchymal stem cells migration and prevents apoptosis. Cell Biol Int..

[36] Moloney TC, Hoban DB, Barry FP, Howard L, Dowd E (2012). Kinetics of thermally induced heat shock protein 27 and 70 expression by bone marrow-derived mesenchymal stem cells. Protein Sci..

[37] Gao F, Hu X, Xie X, Xu Q, Wang Y, Liu X (2010). Heat shock protein 90 protects rat mesenchymal stem cells against hypoxia and serum deprivation-induced apoptosis via the PI3K/Akt and ERK1/2 pathways. J Zhejiang.

[38] Moya A, Larochette N, Paquet J, Deschepper M, Bensidhoum M, Izzo V, Kroemer G, Petite H, Logeart-Avramoglou D (2017). Quiescence preconditioned human multipotent stromal cells adopt a metabolic profile favorable for enhanced survival under ischemia. Stem Cells..

[39] Saxton RA, Sabatini DM (2017). mTOR signaling in growth, metabolism, and disease. Cell..

[40] Galleu A, Riffo-Vasquez Y, Trento C, Lomas C, Dolcetti L, Cheung TS, von Bonin M, Barbieri L, Halai K, Ward S, Weng L, Chakraverty R, Lombardi G, Watt FM, Orchard K, Marks DI, Apperley J, Bornhauser M, Walczak H, Bennett C, Dazzi F (2017). Apoptosis in mesenchymal stromal cells induces in vivo recipient-mediated immunomodulation. Sci Transl Med.

[41] Chang C-L, Leu S, Sung H-C, Zhen Y-Y, Cho C-L, Chen A, Tsai T-H, Chung S-Y, Chai H-T, Sun C-K, Yen C-H, Yip H-K (2012). Impact of apoptotic adipose-derived mesenchymal stem cells on attenuating organ damage and reducing mortality in rat sepsis syndrome induced by cecal puncture and ligation. J Transl Med..

[42] Bustos ML, Huleihel L, Meyer EM, Donnenberg AD, Donnenberg VS, Sciurba JD, Mroz L, McVerry BJ, Ellis BM, Kaminski N, Rojas M (2013). Activation of human mesenchymal stem cells impacts their therapeutic abilities in lung injury by increasing interleukin (IL)-10 and IL-1RN levels. Stem Cells Transl Med..

[43] Hong Y, Kim Y-S, Hong S-H, Oh Y-M (2016). Therapeutic effects of adipose-derived stem cells pretreated with pioglitazone in an emphysema mouse model. Exp Mol Med..

[44] Wang Q, Shen C, Zhu H, Zhou W, Guo X (2013). N-acetylcysteine-pretreated human embryonic mesenchymal stem cell administration protects against bleomycin-induced lung injury. Am J Med Sci.

[45] Yang Z, Concannon J, Ng KS, Seyb K, Mortensen LJ, Ranganath S, Gu F, Levy O, Tong Z, Martyn K, Zhao W, Lin CP, Ma G, Karp JM (2016). Tetrandrine identified in a small molecule screen to activate mesenchymal stem cells for enhanced immunomodulation. Sci Rep.

[46] Zhao Y-F, Xiong W, Wu X-L (2014). Mesenchymal stem cell-based developmental endothelial locus-1 gene therapy for acute lung injury induced by lipopolysaccharide in mice. Mol Med Rep..

[47] Martínez-González I, Roca O, Masclans JR, Moreno R, Salcedo MT, Baekelandt V, Cruz MJ, Rello J, Aran JM (2013). Human mesenchymal stem cells overexpressing the IL-33 antagonist soluble IL-1 receptor-like-1 attenuate endotoxin-induced acute lung injury. Am J Respir Cell Mol Biol..

[48] He H, Liu L, Chen Q, Liu A, Cai S, Yang Y, Lu X, Qiu H (2015). Mesenchymal stem cells overexpressing angiotensin-converting enzyme 2 rescue lipopolysaccharide-induced lung injury. Cell Transplant..

[49] Chen H, Xiang H, Wu B, Zhang X, Li M, Liu J, Li J, Ren Z, Du B, He K, Zeng Q, Yang C (2017). Manganese superoxide dismutase gene modified mesenchymal stem cells attenuates acute radiation-induced lung injury. Hum Gene Ther.

[50] Liang OD, Mitsialis SA, Chang MS, Vergadi E, Lee C, Aslam M, Fernandez-Gonzalez A, Liu X, Baveja R, Kourembanas S (2011). Mesenchymal stromal cells expressing heme oxygenase-1 reverse pulmonary hypertension. Stem Cells..

[51] Chen Y-B, Lan Y-W, Chen L-G, Huang T-T, Choo K-B, Cheng WTK, Lee H-S, Chong K-Y (2015). Mesenchymal stem cell-based HSP70 promoter-driven VEGFA induction by resveratrol alleviates elastase-induced emphysema in a mouse model. Cell Stress Chaperones..

[52] Granton J, Langleben D, Kutryk MB, Camack N, Galipeau J, Courtman DW, Stewart DJ (2015). Endothelial NO-synthase gene-enhanced progenitor cell therapy for pulmonary arterial hypertension: the PHACeT Trial. Circ Res..

